# Ex-opthalmic Goitre, or Grave’s Disease

**Published:** 1872-03

**Authors:** J. W. Southworth

**Affiliations:** Toledo, Ohio


ART. IV.—*Ex-opthalmic Goitre, or Grave's Disease.* Reported by J. W. SOUTHWORTH, Toledo, O.

Mrs. J. H., aged 35. Nervous temprement. Habits of life temperate; has worked hard late years making beaver overcoats. Has one child, a girl of 5 summers. Had occasional "rush of blood to head" in 1867, with vertigo; cold water relieved it. Had slight tremor of lips and palpitations of heart when over worked or excited. After a hard week's work felt a sense of general weakness and increased trembling of voluntary muscles. Palpitation of heart soon became more or less persistent. In latter part of 1867, first noticed enlargement of thyroid gland; protrusion of eyes soon followed, and slight impairment of vision, with a wild staring look. These symptoms were more or less troublesome until 1868, when œdema of lower extremities appeared, but was not inconvenient unless she worked long, standing up; in the morning it was absent. (Had œdema with first child, 5 years ago, recovered soon.) In the autumn of 1868 was attacked with diarrhœa, and has not been free from it since; sometimes having six or eight passages in twenty-four hours, and was often obliged to get up two or three times during the night. Excessive lachrymation, loss of appetite, irritability of stomach and bladder, with headache and fever soon followed, the latter being worse at night. Tenderness, with radiating neuralgic pains in intercostal spaces in front was also present; and profuse bronchorrhœa, (muco-purulent, and sometimes streaked with blood,) attended with emaciation and inability to sit up long in bed.

On January 1st, 1869; when she first came under the care of Dr. N. H. Kimball, Adrian, Mich.,(with whom the writer was then associated,) we found inordinate action of heart, each beat notably jarring the patient's *entire body;* pulse 140 to 150, soft and quick. Area of apex beat larger than natural, very forcible, and felt out side of linea mammalis. A marked thrill felt over both carotids high up in the neck; a notable leaping up of the vessels at each pulsation,accompanied turgescence and pulsation of external jugular and superficial veins.

Superficial cardiac dullness extending to left nipple, the latter overlying the fourth rib, as in the male, from atrophy of mammary glands.

A loud blowing systolic murmur, heard loudest at base of heart, transmitted up the carotids as loud, if not louder; heard also at apex, but not conveyed to the left, nor heard at lower angle of scapula, or in the inter-scapular space.

Respiratory murmur very distinct in all parts of chest, and accompanied by coarse mucous rales on both sides. (Other symptoms as previously stated as existing in 1868.)

The enlargement of thyroid gland was soft and easily diminished by pressure, and equalled an ordinary lemon in size. Sputa offensive in odor; evacuations of bowels occasionally bloody. Diagnosis Ex-opthalmic Goitre, or Grave's Disease. Prognosis not very flattering either to the friends or physicians.

She was placed under the influence of Veratrum V. and Foxglove to control the circulation. Quinine and strychnia, with comp. spts. of lavender to strengthen the shattered nerves. Also a laxative and tonic powder of podophyllin, nit. of potassa and carb. of iron was given. The powder, however, was omitted on second or third day on account of salivation, induced, we suppose, by the podophyllin, as there was *no calomel given.* Animal broths, eggs, etc., for diet Mustard sinapisms were applied to chest and the tenderness and pains got better. At the solicitation of her husband teaspoonful doses of rye whiskey and rock candy were allowed three or four times a day, with apparent benefit.

However, from the above combined treatment she rapidly improved, so that in six weeks she was able to walk, all the symptoms having notably subsided, the cough and bronchorrhœa disappearing together The appetite restored, irritability of stomach, &c., gone also.

In two months she began to work some around the house, and in the course of three or four months she was quite fleshy and of good color. The heart's action was then, (without medicine) 100 to 110 per minute; respiration, 23; cardiac murmus faint. No cough or expectoration; turgescence and pulsation of veins absent; thyroid gland diminished to size of a walnut. Has occasional, but slight, muscular tremors, and only after undue exertion or excitement. Œdema of lower extremeties almost gone.

The treatment was not materially changed throughout. The veratrum was omitted the second or third week. Bismuth and

Dover's Powder combined with rhei. and soda was given to control the diarrhœa. Oxide of zinc was added to the foxglove and spts. of lavendar mixture after the veratrum was omitted. We gave the foxglove as a *cardiac sedative,* believing we should *realize* its so called tonic action on the heart *indirectly, i. e.* stopping the heart from exhausting itself by such rapid and forcible action. The patient is still in good health, and in spite of our protestations, resumed her avocation as soon as she could run a sewing machine. The urine was tested for albumen but none was found.

Present condition: " My health has been very good the past year; have a little numbness of fingers, and dizziness occasionally; some headache; no diarrhœa nor cough. The bunch in my neck enlarges a little when I don't feel well as usual. My feet bloat some." Her pulse is 95, and respiration 20 per minute. Has not taken medicine in almost two years.

